# “Psychiatric emergency consultations of minors: a qualitative study of professionals’ experiences”

**DOI:** 10.1186/s12888-024-05996-6

**Published:** 2024-08-07

**Authors:** P. So, LA. Nooteboom, RM. Vullings, CL. Mulder, RRJM. Vermeiren

**Affiliations:** 1 Youz, Center for Youth Mental Healthcare, Rotterdam, the Netherlands; 2 Parnassia Psychiatric Institute, Rotterdam, the Netherlands; 3 Erasmus MC, Epidemiological and Social Psychiatric Research Institute, Department of Psychiatry, University Medical Center, Rotterdam, the Netherlands; 4 Department of Child and Adolescent Psychiatry, LUMC Curium, Leiden University Medical Center, Leiden, the Netherlands; 5 Faculty of Medicine, Leiden University, Leiden, The Netherlands

**Keywords:** Emergency psychiatry, Minors, Professionals’ experience, Parents, Psychiatric emergency admission

## Abstract

**Background:**

Psychiatric emergency assessment of minors can be a complex process, especially for professional staff who are not specifically trained in handling child and adolescent emergency patients. As minors cannot usually express their feelings and experiences as well as adults, it is difficult to form an accurate picture of their condition and to determine what kind of emergency care is needed, for instance whether or not a psychiatric emergency admission is necessary. We lack insight in what professionals at emergency departments need to adequately assess these minors and their families. The aim of this study was to explore staff members’ experiences with assessing minors and explore recommendations for improving their ability to provide appropriate support.

**Methods:**

Guided by a topic list with open-ended questions, we conducted 11 semi-structured interviews with staff working at psychiatric emergency services. Thematic analysis enabled us to identify five main themes: (1) young age and the crucial role of parents; (2) professionals’ feelings, especially uncertainty; (3) psychiatric emergency admissions and the alternatives to them; (4) regional differences in organization and tasks; and (5) options for improving care.

**Results:**

The staff interviewed all agreed that it was often complicated and time consuming to take full responsibility when assessing minors with serious and urgent psychiatric problems. Most found it difficult to determine which behaviors were and were not age-appropriate, and how to handle systemic problems during the assessment. When assessing minors and their families in crisis, this led to uncertainty. Professionals were especially insecure when assessing children under age 12 and their families, feeling they lacked the appropriate knowledge and routine.

**Conclusion:**

Customized expertise development and improved regional embedding of the psychiatric emergency service in the child and adolescent services will reduce professionals’ uncertainty and improve psychiatric emergency care for minors.

**Supplementary Information:**

The online version contains supplementary material available at 10.1186/s12888-024-05996-6.

## Introduction

Over recent years, the number of minors experiencing a psychiatric crisis has increased worldwide [e.g. 1, 2]. More specifically, suicidal ideation and behavioral problems have increased significantly in minors presenting at psychiatric emergency services [3].

For several reasons, it is often difficult during a psychiatric emergency assessment to determine what kind of help is needed by a minor in crisis. Acute mental health problems in childhood occur at the interface of trauma, developmental problems, problematic parenting, systemic problems, behavioral problems, learning problems, and/or somatic problems. Such conditions are frequently complicated by family disruption, suicidality, aggression, and risky impulsive behavior that all require containment and de-escalation [4]. Neither can minors always express their feelings and experiences as adults do, which often makes it difficult to gain an accurate picture of their condition [5].

In most countries, children and adolescents in crisis present to emergency departments (EDs) of general or pediatric hospitals, were a trained ED nurse or physician serves as pediatric emergency care coordinator. However, in most regions of the Netherlands, outpatient psychiatric emergency services have round-the clock responsibility for assessing patients of all ages who are referred to them with acute psychiatric problems. Staff at these services comprise community psychiatric nurses, physicians, and psychiatrists, all of whom have been trained in the diagnosis and treatment of adult emergency patients, but not specifically in handling child and adolescent emergency patients. Like earlier authors [6–8] we received signals from those in practice that professional staff often struggle when assessing minors in crisis. This study was therefore intended to gain insight into the difficulties they experience.

Few studies, all conducted in the USA, have investigated the perspectives of staff at EDs, either on the assessment of minors in psychiatric emergencies, or on what is needed to optimize care in this target group. Dolan & Fein found that the problems faced by healthcare professionals in the emergency mental healthcare of children and adolescents included organizational problems such as the shortage of inpatient beds and of pediatrically trained mental health specialists [6]. A qualitative study by Bowden et al. found that three themes were related to the challenges of assessing pediatric patients who had attempted suicide attempts or had suicidal ideation: the interviewees in question felt ill-equipped to provide appropriate care for this specific patient group in the ED; were frustrated by the poor availability of inpatient beds; and often felt helpless or intense despair when more and younger children with serious suicidality or agitation presented at the ED [7]. Another qualitative study by Foster et al. found that, due to their perceptions of insufficient training and experience, ED staff felt an overarching moral distress, especially when assessing agitated minors. They also voiced frustration about the barriers they faced when referring children and adolescents to specialized inpatient or outpatient care. In their experience, the prolonged ED boarding of minors had negative impacts on the care of other ED patients [8].

Surprisingly, none of the studies above referred to the role of parents or caregivers in the interviews, or how their involvement affected the assessment. As parents play an important role in minors’ lives, this is striking. Usually, when a minor is assessed, it is necessary to involve at least one family member. First, they can provide information. Second, separately and together with the minor, they can themselves be assessed for their pedagogical skills and ability to manage the minor’s behavior. Usually, an emergency service visit by a minor is the result of a support-system breakdown, in which parents, teachers, or group homes conclude that their ability to deal with a minor’s behavior has been overwhelmed [9]. In such situations, they often wish to know whether a psychiatric emergency admission can be arranged. While hospitalization may be unavoidable for a minor who has active suicidal ideation with intent and plan, the risk for suicide is known to increase significantly immediately after discharge [10].

Most professionals agree that psychiatric problems in minors are best managed in the home setting, where systemic interventions can take place directly within a child’s environment [e.g. 11, 12]. In addition, as most countries have reduced the number of child and adolescent psychiatric inpatient beds [e.g. 13, 14], it is preferable that crises are resolved without hospitalization. Whether or not a minor is hospitalized can depend, at least in part, on the presence or absence of parents at the emergency assessment [15] and on the degree of disruption within the family [16].

In the Netherlands, another factor adds to the importance of the family’s or caregiver’s involvement in emergency assessment and treatment: under Dutch law, parents play an important role in determining what can be done to manage crisis situations involving minors under 16. Since the options are best sought within the support system, the parents and the professionals must agree on what must be done to ensure safety, and hopefully to resolve the crisis. For all the reasons mentioned above, involving parents is important when assessing minors in crisis, but we do not know how the staff at the psychiatric emergency services handle this during the emergency consultation.

In this qualitative study, we aimed to gain insight into the experiences of the staff at the psychiatric emergency services in the Netherlands, who assessed minors and their systems, and to learn more about what they believed to be necessary to optimizing care. Considering they have had no specific training in child and adolescent psychiatry, we hoped that insight into their experiences would enable us to identify and evaluate the challenges they faced when attempting to resolve the crises minors undergo during a psychiatric emergency.

## Methods

To report on the findings of semi-structured interviews with staff members of two outpatient psychiatric emergency services in the Netherlands, we chose a qualitative methodology that would elicit insight into the phenomenon within its own specific context [17]. The study was conducted in accordance with the consolidated criteria for reporting quality research guidelines [COREQ, 18]. After assessing the study, the Medical Ethics Review Board at Leiden University Medical Center stated that the research in question was not subject to the Medical Research Involving Human Subject Act (non-WMO approval number: N23.068).

### Setting

The context for this study was two outpatient psychiatric emergency services in the Netherlands. Part of an integrated mental healthcare institution, they offer round-the-clock assessment and treatment of people of any age who are in severe behavioral and emotional distress, whom they help to stabilize, and, when indicated, whom they refer to other types of care (e.g., outpatient or inpatient treatment). Most patients are referred to the emergency service by telephone – by general practitioners (GPs), mental healthcare workers, police, and the EDs at general hospitals. On the basis of the information obtained by telephone, a trained healthcare professional – a so-called triagist – determines whether an acute face-to-face assessment is necessary. According to the situation, this can take place at home, in the hospital or at a dedicated venue. In emergency situations, patients are examined wherever they are by two mental healthcare professionals: a community psychiatric nurse and either a psychiatrist or a physician working under supervision of a psychiatrist. As, in the Netherlands, minors represent less than 5% of the emergency patients in these services, they are assessed by specialists with a background in psychiatric emergency medicine who work with patients of all ages, not specifically minors. Most minors seen for emergency consultation are adolescents aged 13 to 18 years, less than 5% of all pediatric psychiatric emergency consultations involve children 12 years and younger. This corresponds to less than 5 children per year per emergency service [19].

### Participants

To be included in this study, participants had to work in an outpatient psychiatric emergency service as a psychiatrist or community psychiatric nurse. For purposes of gaining a broad perspective and gathering all viewpoints and issues relevant to the various subgroups, recruitment took place through purposeful maximum variation sampling [20]. We wished to the examine as fully as possible four main attributes: the region of the emergency service, the participants’ sex and profession, and their active years of employment in the psychiatric emergency service. Team managers within two large psychiatric emergency services were approached and asked to distribute the study’s recruitment letter. Potential participants were contacted directly by the first author (PS) in an email message that provided information on the study and planned an interview at the site of the emergency service. Before the interview, these participants were asked to sign for informed consent.

### Data collection

Semi-structured interviews were conducted between September and November 2023 by the first author (PS, female) and a medical student (RV, female). The interview was developed for this study, for its content please see supplementary interview professionals, English version. These interviews were intended to gain insight into the experiences of those staff at the psychiatric emergency services who assessed minors with a broad range of acute psychiatric problems including, but not limited to suicidality, and to learn more about what they believed to be necessary to optimizing care. The interviews were guided by a topic list with open-ended questions; based on previous literature [2, 6–8, 16, 19, 21, 22]. The topic list was then supplemented by inputs from two preliminary interviews with a psychiatrist (female) and a physician (male), both of whom had over five years of employment at the psychiatric emergency service. During the interview process, this list was also supplemented by inputs from the reflexive meetings of the researchers held after each interview.

Per interviewee, we noted the sex, profession, length (in years) of their active employment in the psychiatric emergency service, their specific experience in child and adolescent psychiatry, and whether or not they were a parent. As well as general questions on the procedure for psychiatric emergency consultations in minors and their systems, the topic list included questions on (1) barriers and facilitators in relation to the interviewee’s ability to assess the interaction with minors, the nature and quality of their collaboration with colleagues, and the organization of care (including preconditions, laws and regulations); (2) the role of parents (or other caregivers) in psychiatric assessments and in resolving crisis situations involving a minor, and how interviewees handled this role during the emergency consultation; and (3) which options the interviewees proposed for improving the help provided to minors with serious and acute psychiatric problems who had been referred to the outpatient psychiatric emergency services.

The interviews were conducted in Dutch, recorded, and transcribed verbatim. Although, with the exception of one psychiatrist (P6), the interviewees had never met the researchers before, they all knew that the first author (PS, a child and adolescent psychiatrist) worked as a trainer of residents in child and adolescent psychiatry and was specialized in psychiatric emergency care. Two psychiatrists mentioned this during the interview, referring to what the interviewer might think about how minors in a psychiatric emergency should be handled.

No interviewee expressed an interest in commenting on the transcripts. Field notes were obtained by the student assistant during the interviews, after which a reflexive meeting took place to evaluate the interview process and discuss new insights. The quotes presented in the result section were translated from Dutch to English by the first and second authors (PS and LN, respectively).

### Analyses

All transcripts were imported into MAXQDA (version 22), a program designed for computer-assisted qualitative data, text, and multimedia analysis. The analytic process was guided by reflexive thematic analysis of the qualitative data [23, 24]. The interviews were transcribed by the first author, who, in parallel with the student, read them all in order to familiarize themselves with the data. After six interviews, early hypotheses and initial coding were developed. So as not to overlook any information, an open coding technique with line-by-line analyses of the transcripts was used. On the basis of the analytical and theoretical ideas developed during the interviews, codes were grouped into categories, and themes were developed. The categories and themes were discussed with the second author (LN), and agreement was reached on the thematic framework. After nine of the eleven interviews had been coded, no additional codes were added, an indication that saturation had been reached and that no supplementary interviews were needed [25].

## Results

### Demographics

A total of five community psychiatric nurses and six psychiatrists were interviewed. Their demographic characteristics are shown in Table 1.

**Table 1:** Professionals’ demographic characteristics

Interviewee	sex	experience PES	parent	worked in CAP
N1	f	>10 years	yes	no
N2	f	3 years	yes	no
N3	f	>10 years	yes	no
N4	f	>10 years	yes	no
N5	f	>10 years	yes	no
P1	f	>10 years	yes	9 months
P2	f	4 years	no	no
P3	m	>10 years	yes	no
P4	f	8 years	yes	2 years
P5	f	>10 years	yes	no
P6	m	2 years	no	no

PES = psychiatric emergency service

CAP = child and adolescent psychiatry

N = community psychiatric nurse, P = psychiatrist

f = female, m = male

### Participants’ descriptions of the situations they faced when assessing minors and their families

All the professional staff interviewed shared the opinion that it was often complicated to take responsibility for assessing not only minors with serious and urgent psychiatric problems, but also their families. They also agreed that this process was more time consuming than in adults.

Thematic analysis reveiled five main themes: (1) young age and the crucial role of parents and caregivers; (2) professionals’ feelings, especially uncertainty; (3) psychiatric emergency admissions and the alternatives to them; (4) regional differences in organization and tasks; and (5) options for improving care. The themes are further explained in the following sections.

#### Young age and the crucial role of parents/caregivers

“In my experience, they’re like two groups. First there are the boys and girls around 15,16,17 who could also have been 18 years old, and who are suicidal or have attempted suicide, but who you can have a normal conversation with afterwards. They also have parents who are more or less involved or more or less normal. Then, of course, you have those kids who are often a bit younger, but where, I think, the problems are not only with them, but more in the system. Difficult parents, parents that don’t want to take their child back, so to speak. Yes, that’s a completely different group than the first group.” P5.

All participants stated that most minors in the emergency psychiatry services were aged between 15 and 18. Just as with adults, their reasons for referral often concerned emotion-regulation problems, suicidality, and self-harm. Independently of a minor’s psychiatric condition, a role was often played by puberty, childrearing problems, and conflicts with their parents. Most professionals found it difficult to determine what was age-appropriate in terms of intense feelings and impulsive and rebellious behavior.

“And the sad thing is that those people, those teenagers, could do really weird things, right? They act much more thoughtlessly in suicide attempts. That you think, where, God, yes, that paracetamol, you know… You see more of that these days, of course. And yes, I do think that these kids don’t actually know what they’re doing. And then they say, I want to die. But they don’t have a clue about any of the possible consequences. So, to me, that’s much more on a primary level than on a level they’ve actually thought about. And very often when you speak to them, they say they don’t really want to die, but that they do feel deeply unhappy.” N3.

Several professionals indicated that suicidality among minors often led to a request for an emergency assessment, because general practitioners and other professionals who refer suicidal minors to the psychiatric emergency services do not dare to take the responsibility. Even with those showing mild signs of suicidality, they seem afraid that a future attempt may succeed, and do not have – or take – the time to talk extensively with the minor and their parents. Parents are sometimes so shocked after a suicide attempt that they do not dare to leave their child alone for a second, but do not dare to talk about what happened either.

“And in my opinion – though you often see this in general practitioners – suicidality stands in the way of the conversation. Meaning that the actual advice is to just talk to someone – which is precisely what parents and GPs want the services to do for them. And because we’re supposed to be the experts, we have to [do it].” N3.

Almost all participants indicated that they preferred to conduct the conversation with parents and minor together, but that they also honored a minor’s wish to talk separately. During the interviews, it became clear that our participants did not have a good grasp of Dutch laws and regulations on who had decision-making power and on the rights of parents and of minors aged 12–16 to information. In practice, however, this did not seem to lead to problems: as safety is often at stake in psychiatric emergency consultations, it is permissible in the Netherlands to breach confidentiality, regardless of age. A professional’s suspicions of danger are always shared with the parents – if, for instance, a minor says that he she is still thinking about or planning suicide.

Almost all participants regarded collaboration with parents as very important, as parents know their child best, and are always needed when drawing up a plan for managing the crisis. Participants often stated that parents were exhausted, wanted to hand over their child, or demanded hospitalization. Some parents were very persistent in their refusal to participate in the assessment or take their child home.

Participants also stated that it took time to build a working relationship with minors and parents alike. They considered it important to manage parental expectations, show understanding, and provide explanations.

“Of course, you have a few parents who don’t contribute to the solution. But they certainly exit. Yes. You’d rather that some of them weren’t involved, or think it would be better if they weren’t there. But that’s just a handful. Most parents want the same: that everything goes well with their child. And they want to do everything they can to achieve it. So, basically, I think parents contribute to the solution. In the best cases, parents really *are* your partner, and you can make a proper plan with them.” P4.

Participants referred to two social factors that appeared to play a role in crisis situations involving minors: increased social media use, and pressure to perform. They also referred to a general trend in families that was potentially important, as it risked undermining the minors’ resilience: families who showed less connection and support, and who communicated less with each other, for example about how to deal with problems or feelings.

“Maybe it’s also a sort of social trend that we want to fix problems by turning to authorities and experts – while the solution may very often lie in just having a conversation. And that, or so it seems to me, is something we’ve become very afraid of. And then, I mean, that’s what we do in our assessments. Because eventually it’s not some kind of list that determines whether you consider someone suicidal or not – it’s about you making contact, about you simply going to see how someone relates to life. And in a manner of speaking, you confront someone – talk to them about how things are going. Anyway, people find that complicated. And if you don’t do it on a daily basis, I can understand that.” P6.

Although children under 12 present much less for emergency assessment, assessing them was seen as more complex. Participants described how challenging it was to make contact with them, and that younger children are often unable to explain what the problem is. This can lead professional staff to feel insecure about the reliability of their assessment. With these children, even more than with older children, they considered the involvement of family or other carers to be of great importance to understanding what was going on.

#### Professionals’ feelings, especially uncertainty

“That’s exactly what I like about the emergency service: you see so many different people – with dementia, or elderly people with psychiatric problems, but also young people. It adds variation to the job – yet another type of problem for you to deal with. It also demands a different way of working, which is what I also enjoy, and also brings all sorts of opportunities for learning.” P5.

Most participants stated that even though they were not generally accustomed to working with minors on a regular basis, assessing minors was simply part of their job. Several nonetheless experienced uncertainties, particularly about missing signals or making the wrong decision because they lacked knowledge and experience. In their training or previous work, most had never specifically encountered child psychiatry. The few who had seemed to be more relaxed about assessing minors as part of their job.

“In the beginning when I came here, young people had their own emergency service – in the daytime, in fact. I think that ended in 2012 or 13, possibly later. It was mainly an organizational and pragmatic choice, I believe – something I thought it was a pity we were burdened with. Purely because I often feel that our usual approach doesn’t work but I don’t know how to fix it. The younger they are, the less we know about our options and the fewer our possibilities for making contact. And yes, I do indeed still think it’s a pity we don’t have [the original system, in which real experts conducted] emergency assessments.” P3.

The uncertainty that arose from their perceived lack of knowledge also complicated participants’ ability to determine their own policy, and, for example, to go against parents’ wishes or to convince hospital bed managers of the need for admission.

“So if there’s any risk to the child, and these parents say, well, we don’t dare to take her home, I’ll make sure we hospitalize. I feel so incapable that I don’t want it on my conscience. I’m really not going to take any risks with that child. So if there are risks and if the parents want hospitalization, I’ll go for hospitalization sooner than I would with an adult in a similar situation.” P2.

The degree of uncertainty also seemed to be related to task perception. Participants who indicated that their main priority was crisis management, and that their task was to conduct short-term safety-focused interventions, were much less likely to describe feelings of incompetence and uncertainty than those who described their task as diagnosing or indicating appropriate psychiatric treatment. Some, especially those who had children themselves, also felt personally affected by these young patients’ situations.

Psychiatrists who did not specifically choose to work for the psychiatric emergency services, but whose mandatory services outside office hours sometimes required them to accept it, were particularly likely to describe negative feelings about also having to assess minors.

“Whenever a child or youngster is referred to me, I really do feel more tense about it. I can also be frustrated by the assessments, as I wonder how the system manages to push kids like this onto people who aren’t the right ones to deal with them – frustrated that I have to do it, or, that I should be responsible for it, all while having the feeling that I’m not the right person for it. So, yes, there’s definitely tension and frustration.” P2.

#### Psychiatric emergency admissions and the alternatives to them

The central question during an assessment is often whether there is an indication for voluntary or compulsory psychiatric emergency admission. Interviewees experience emergency admission as very drastic. Due to its potentially negative influences within the greater group in an acute ward, it also risks exacerbating any symptoms. Most participants agreed that little treatment is provided during an emergency hospitalization, and when it ends, the minor simply returns home to a situation that is unchanged.

“Of course, you often hear that admission is counterproductive, especially when a child has a possible developmental disorder on top of puberty. They then arrive in a ward with other kids who have… well, quite a lot of bad stuff behind them, who can help you pick up some pretty bad behavior.” N5.

Although most participants agreed that there was a shortage of child and adolescent inpatient beds, emergency hospitalization is usually possible, even though it may take a long time to arrange it and even though the clinic in question may be far away. Rather than more beds, the participants felt that alternatives such as Intensive Home Treatment (IHT) and child-friendly time-out beds would be better alternatives for minors requiring emergency hospitalization.

“Personally, I’d argue for something we once had for a very short period – a project called IHT Youth. Coincidentally, we were taking about it just the other day. And I had little experiences with that because they had limited capacity and limited options. But that was something that could actually have been expanded a bit – I think it offered more than just hospitalizing kids because they could stabilize at home. We have IHT for adults, and it works perfectly. Something like that for kids? That would really be very desirable. You could then intervene sooner, so it wouldn’t endlessly go on simmering at home. I definitely see added value.” N4.

*4. Regional differences in organization and tasks.*

The tasks of the psychiatric emergency services differed between the two regions represented in this study. One difference concerned whether or not regular child and adolescent psychiatry consultations inside the hospital were a task of the emergency services. Over the years, various agreements had also changed. These differences could create ambiguity, which some participants found stressful.

“I also think we experience a greater burden these days. Back when the emergency services worked at a more local level, you knew the region, and if you had to assess a minor, it was once every three months or so. But now, of course, we’ve thrown everything onto one big heap […]. And because of the differences between the regions, there’s more uncertainty.” P1.

The participants all indicated that it would help if they knew more about what kind of support was available for minors and their families in their region. The triage nurse seemed to know most about the regions, tasks, options, and contact persons. Whenever a triage nurse had not been involved in assessments, participants reported having had less insight into the social services youth directory. Similar situations arose when psychiatric emergency staff dealt with child and adolescent emergency consultations exclusively outside office hours. Participants found it important to have an easily accessible child psychiatrist as a backup, and missed them when they were unavailable. Most psychiatrists and some community psychiatric nurses were critical of child and adolescent psychiatrists who did not want to provide services outside office hours, even for their own patients.

“When parents of kids who are being treated by [instituter’s name withheld] call the out-of-hours GP service for a phone consultation, they get referred to us. I think that’s really ridiculous – we have to call them back, and eventually we have to see them, and eventually we have to do all sorts of things, all while we have no patient file. So, one day, I, like, called the institute in question and said, call these parents yourself. See what they need. They’re your patients, so take care of them. When they told me that’s not the deal, I said maybe not, but I don’t care, as it’s certainly a better way of caring for your patients.” N1.

#### Possibilities for improving care

Most participants wished for further training in child and adolescent psychiatry – training that would be appropriate to the complex task of assessing minors in daily practice. They suggested holding meetings in which, together with child and adolescent specialists, staff at the psychiatric emergency services could reflect on cases involving minors, and could get feedback on their decisions. These meetings might increase their knowledge of possible alternative interventions and reduce the insecurity they felt. They should also include topics such as emergency medication, short systemic interventions, and the social services youth directory.

“Anyway, I’d like us to have occasional lessons on cases, or just to have case discussions. They don’t have to be regular, but it would be very valuable to reflect on different cases, especially if we don’t have much expertise in them. Because you don’t get much feedback on that. Of course, somewhere in the system, there are also other files on a patient. So, if I have [an adult] hospitalized here, I look at their file after a few days to see how they’re doing. But if I admit a minor… then I lose them. And you don’t get any feedback on whether it was a good intervention or not.” P6.

To further reduce professional staff’s uncertainty, a clear overview of the tasks of the psychiatric emergency service should be available per region. This should also include a list of the options and contact persons needed to organize emergency care for minors, including an easily accessible child psychiatrist as a backup. Participants mentioned that while triagists could fulfill a role in this, getting to know other professionals when working on cases would increase mutual understanding and improve collaboration.

“I think it’s also very important that you yourself know the social services directory, or that your colleagues do – that you know how things work here – and with kids, of course, there are all kinds of youth care services, aren’t there? I don’t actually know what they do or when one particular service does this or the other does that. Because I also hear, you know, that things sometimes get pushed towards an out-of-home placement […] when children have mental trouble thanks to… well, a distorted environment. And I don’t know how all that works, or who I should contact.” P2.

## Discussion

This study was intended to gain insight into the experiences of those working as psychiatric emergency staff who evaluated minors with serious and urgent psychiatric problems, and also evaluated their families. In interviews with community psychiatric nurses and psychiatrists working in outpatient psychiatric emergency services, we learned a great deal about their first-hand experiences.

The interviewees, who were not specifically trained to handle child and adolescent emergency patients, described several challenges they faced when assessing minors in crisis. Many professionals mentioned that taking responsibility in the assessment of suicidality in minors is experienced as complicated, also due to the anxiety of the parents. However, the focus of this study was broader than suicidality and yielded other topics as well. Most found it difficult to determine what was age-appropriate behavior and how to handle systemic problems during the assessment. They felt especially insecure when assessing children under 12 and their families, an activity for which they felt they lacked knowledge and routine. Adding to these insecurities were differences between regional healthcare organizations and the fact that most of them had little knowledge of the social services youth directory. Although they acknowledged a shortage of child and adolescent inpatient beds, it was not regarded as very problematic. A bigger problem was the lack of alternatives (such as IHT) for psychiatric emergency admissions.

The results of our interviews with professional staff working at outpatient psychiatric emergency services are consistent with previous findings in studies on the perspectives of the ED staff, who, as well as shortages of pediatrically trained mental health specialists, also referred to emotional challenges [6–8]. In our study, however, parents were an entirely new topic, particularly with regard to the ways in which staff evaluate and handle their role in assessing minors during a psychiatric emergency. Collaboration with parents is not only crucial, it is also challenging: parents are often exhausted, and sometimes pressure emergency-service clinicians to have their child admitted to a psychiatric hospital.

The experiences of our interviewees enable us to conclude that it takes time and persuasiveness to manage expectations, show understanding, and provide explanations – and that there is still a risk that parents will not feel helped by an assessment that results in referral to outpatient care. They often focus their hopes on the possibility that the psychiatric emergency service will come up with a solution, and thereby overlook the likelihood that problems that have been present for some time will not be solved immediately. More importantly, a one-off emergency assessment provides no basis for entering into the therapeutic relationship necessary for supporting these families adequately. It is an intensive process to involve parents properly: they feel powerless and have sometimes lost confidence in help [26]. The question is which role emergency care professionals can play in this, and which role lies with the care provider who is involved with the family over a longer period of time.

In the Netherlands there are major regional differences in the organization of psychiatric emergency care for minors. Most regions have an outpatient psychiatric emergency service that is responsible 24/7 for assessing patients of all ages with acute psychiatric problems. Some regions have a specialized psychiatric emergency service for minors during office hours, while others also provide coverage outside them. It is therefore possible that some psychiatrists have been trained in a region where adult emergency services never assessed minors, thus reinforcing their feeling that assessing minors is not inherent to their work – which, of course, can make them feel more insecure.

Since 2022, a mandatory child and adolescent psychiatry internship has been included in psychiatry training in the Netherlands. Given the regional differences and the complexity of the cases seen during psychiatric emergency consultations, our interviewees had various opinions on whether this would solve such feelings of uncertainty. Most stated that it was important that supervised psychiatric emergency assessment of minors becomes a structural part of this internship, which should also include case-based discussions and practice focused on system-oriented assessment. Future studies should evaluate the effect of the addition made to the psychiatry training in 2022.

### Practical implications

To improve psychiatric emergency care for minors, we explicitly asked the participants in our study about their needs with regard to feeling better equipped for this part of their job. Our analysis of the interviews leads us to believe that uncertainties of the type they referred to would be reduced by customized expertise development and by improving the regional embedding of the psychiatric emergency services in the local child and adolescent services, with an easily accessible child psychiatrist as a backup. The use of a rating scales to assess suicide risk in children and adolescents maybe another helpful addition. While no rating scale can predict outcomes with 100% accuracy, they can provide professionals with information that may complement their assessment.

While it is beyond the scope of this study, another recommendation is crucial: to focus on creating the conditions that reduce the need for ED admissions. This would involve a major task for society as a whole. The participants in this study argued the need for more connection between parents and their children, and for more social support in neighborhoods. In families and neighborhoods alike, better social support will increase the capacity of minors and their families to deal with the problems of life [27–29], thereby reducing suicidal ideation and the need for psychiatric emergency consultation. Both nationally and internationally, this is currently consistent with the views of many policymakers: prevention, not cure, should be the primary policy goal. As well as positive relationships within families, between peers, and in the community, mentally friendly education and the reduction of adverse experiences are all important components of a mentally prosperous nation [30, 31].

### Strengths and limitations

Our study provides useful insights into professionals’ experiences with psychiatric emergency consultation of minors and their families. In semi-structured interviews based on a topic guide that included a range of open discussion points, professionals who worked for the psychiatric emergency services were able to share their experiences and opinions in depth. The structured analysis procedure and the reflexive meetings made it possible to thoroughly explore this rich information, while reducing the risk of researchers’ subjectivism [32].

As the organization of psychiatric emergency care is constantly developing, it is important to interpret the findings in their current context. One limitation of our study concerned the possibility of selection bias. The first interviewees had strong opinions about psychiatric emergency assessment of minors by the psychiatric emergency services. During these interviews and the subsequent reflexive meetings, we developed hypotheses and explanations. To check our assumptions and prevent sampling bias, we approached professionals who had not at first responded to our invitation. A subsequent round of interviews added the nuance that had been lacking in the first round.

A second limitation is that the veracity of the participants’ responses may have been affected by the fact that they knew that the first author worked as a trainer of residents in child and adolescent psychiatry. A third limitation resulted from the regional differences we found, in the sense that the transferability of our findings may have benefited from interviewing professionals drawn from more than two psychiatric emergency services with a wider geographic spread across the Netherlands. Future studies might include the experiences of professionals working for psychiatric emergency services in other regions, and of psychiatrists whose training is more recent, and has thus included the mandatory child and adolescent psychiatry internship. Finally, it would have been useful to include the use of rating scales during the assessments in the topic list.

## Conclusion

By interviewing professional staff on their experiences with psychiatric emergency consultation in minors, we wished to contribute to the development of emergency care for minors with serious and urgent psychiatric problems that is of high quality and fully supportive of the patients and their families, but also of the professionals who provide it. Our results indicate that customized expertise development and improved regional embedding of the psychiatric emergency service in the child and adolescent services will reduce professionals’ uncertainty when assessing minors in a psychiatric emergency.

### Electronic supplementary material

Below is the link to the electronic supplementary material.

Supplementary Material 1

## Data Availability

The dataset of interviews in Dutch generated and analyzed during the current study were used under license for the current study and are not publicly available but are available from the corresponding author on reasonable request.

## Abbreviations

ED: emergency department
COREQ: consolidated criteria for reporting quality research guidelines
GP: general practitioner
PES: psychiatric emergency service
CAP: child and adolescent psychiatry
IHT: Intensive Home Treatment
