# Supplementary material for: “Psychiatric emergency consultations of minors: a qualitative study of professionals’ experiences”
Source: BMC Psychiatry. 2024 Aug 7;24:549. doi: 10.1186/s12888-024-05996-6 (PMC11304710; doi:10.1186/s12888-024-05996-6)
Supplement: Supplementary file 1 — Supplementary Material 1 [file 12888_2024_5996_MOESM1_ESM.docx]

Format interview professionals (English version)

| Interviewer introduces herself | Thank you very much for allowing us to schedule this interview. I will first introduce myself briefly. My name is Pety So and I am a child psychiatrist at the Youz emergency clinic in Rijnmond. I am very interested in improving care for young people with serious and acute psychiatric problems. So far, I have done research into the characteristics of minors seen by the psychiatric emergency services. The interviews that I am now going to conduct form the basis of an article that I want to write about the experiences of the staff at the psychiatric emergency services with acute psychiatric assessments of minors. |
| --- | --- |
| Interviewee introduces him/herself | The audio recording is not on yet, so could you please introduce yourself briefly? |
| Emphasize that interview data is processed anonymously | The data I collect during this interview is processed anonymously, i.e. without your name or personal data. |
| Explain the duration, purpose and content of the interview | The interview lasts approximately one hour. If you need a break, this is always possible. If I must ask a question again or explain it again, please say so, this is no problem. We want to find out which factors are experienced as hindering or helping in the acute psychiatric assessments of children and adolescents referred to the psychiatric emergency service and more specifically how the role of parents in these assessments is perceived. This can help to improve psychiatric care for children and adolescents in crisis. You can think of characteristics of the young people, of the parents, but also of the organization of care. So, I have quite a few topics that I want to cover in an hour, so I will have to pay attention to the time and sometimes move on to the next topic a little faster. |
| Request permission for audio recording | It was also stated in the consent form, but are you okay with this interview being recorded? Then I now turn on the voice recorder. |
| Characteristics | I would first like to know some basic information from you:   - Sex, age, parent. - What is your profession? - How many years of experience at psychiatric emergency service? - Did you ever work in child- and adolescent psychiatry? |

**Content of interviews**

The interviews are guided by a topic list with open-ended questions based on factors already known from literature and practice that influence the acute assessment of minors with psychiatric problems in crisis. The topic list was supplemented by inputs from two preliminary interviews with two professionals both of whom had over five years of employment at the psychiatric emergency service. During the interview process, this list was also supplemented by inputs from the reflexive meetings of the researchers held after each interview.

**Opening questions:**

Both in literature and in practice, an increase in children and adolescents with acute psychiatric problems is noticed. Do you recognize that? Do you have the idea that more minors are also being reported to your psychiatric emergency service for an assessment? How does that affect your work? Positive or negative. This can be practical, but also emotional, for example. Can you describe how an acute assessment works, from referral to completion? How do you view the role of parents in these assessments? What could be helpful to facilitate the assessment of children and adolescents? What do you think is needed to improve care?

**Topic list**

**The topics that have already been discussed in reaction to the opening questions do not have to be asked again. The topics that have not yet been discussed will be further questioned according to the list.**

| **Child and family factors** | **Source** |
| --- | --- |
| Age and sex of child | 1, 7, practice |
| Type of problems | 1, 6, 7, preliminary interviews |
| Role of parents | 4, 5, practice |
| Parents present at assessment | 4, practice |
| Together and / or apart | practice |
| Communicating with parents | practice |
| Available network | practice, preliminary interviews |

| **Professional** | **Source** |
| --- | --- |
| Perceived differences with assessments in adult patients. | practice |
| Emotional factors | 1, 3, practice |
| Sense of competence | 1, 2, practice |
| Indications for emergency admissions | 5, 7, practice |
| Training in child and adolescent psychiatry | preliminary interviews |
|  |  |

| **Organization and society** | **Source** |
| --- | --- |
| Laws and regulations | 5, 7, practice |
| Availability of inpatient beds | 1, 2, 3, practice |
| Arranging follow-up care | 3, practice, preliminary interviews |
| Cooperation with other parties | Practice, preliminary interviews |
| Impact COVID-19 | 8, practice |
| Transport and location of assessment | 5, practice preliminary interviews |
| Regional differences | practice, preliminary interviews |

| **End of interview** |  |
| --- | --- |
| Discuss whether the person interviewed has any questions or additional comments. | Do you have any questions or comments or things you would like to add? You can also email to me afterwards. |
| Ask whether the interviewee is interested in the final report. | The results of the interviews are processed into an article. Would you like to receive this article? |
| Thanks for participating | Thank you very much for participating in this interview. |

Literature:

1. Bowden CF, True G, Cullen SW, Pollock M, Worsley D, Ross AM, Caterino J, Olfson M, Marcus SC, Doupnik SK. Treating Pediatric and Geriatric Patients at Risk of Suicide in General Emergency Departments: Perspectives From Emergency Department Clinical Leaders. Ann Emerg Med. 2021 Nov;78(5):628-636. doi: 10.1016/j.annemergmed.2021.04.025. Epub 2021 Jul 1. PMID: 34218952; PMCID: PMC8546759.

2. Dolan MA, Fein JA, Shaw KN, Ackerman AD, Chun TH, Conners GP, Dudley NC, Fuchs SM, Moore BR, Selbst SM, & Wright JL. Technical report - Pediatric and adolescent mental health emergencies in the emergency medical services system. Pediatrics (2011). 127(5), e1356-e1366. https://doi.org/10.1542/peds.2011-0522

3. Foster AA, Sundberg M, Williams DN, Li J. Emergency department staff perceptions about the care of children with mental health conditions. Gen Hosp Psychiatry. 2021 Nov-Dec;73:78-83. doi: 10.1016/j.genhosppsych.2021.10.002. Epub 2021 Oct 15. PMID: 34687946.

4. So P, Wierdsma AI, Kasius MC, Cornelis J, Lommerse M, Vermeiren RRJM, Mulder CL. Predictors of voluntary and compulsory admissions after psychiatric emergency consultation in youth. Eur Child Adolesc Psychiatry 30, 747–756 (2021). <https://doi.org/10.1007/s00787-020-01558-9>

5. So P, Wierdsma AI, van Boeijen C, Vermeiren RR, Mulder NC. Gender differences between adolescents with autism in emergency psychiatry. Autism. 2021 Nov;25(8):2331-2340. doi: 10.1177/13623613211019855. Epub 2021 Jun 3. PMID: 34080460.

6. So P, Wierdsma, AI, Vermeiren RRJM, Mulder CL. Psychiatric Emergencies in Minors: The Impact of Sex and Age. Pediatric Emergency Care 38(6):p 258-263, June 2022. | DOI: 10.1097/PEC.0000000000002674

7. So P, Wierdsma AI, Mulder CL, Vermeiren RRJM. The impact of the COVID-19 pandemic on psychiatric emergency consultations in adolescents. BMC Psychol. 2023 Apr 6;11(1):101. doi: 10.1186/s40359-023-01085-7. PMID: 37024890; PMCID: PMC10078013.
